# The ecotoxicological consequences of microplastics and co-contaminants in aquatic organisms: a mini-review

**DOI:** 10.1042/ETLS20220014

**Published:** 2022-08-16

**Authors:** Farhan R. Khan, Ana I. Catarino, Nathaniel J. Clark

**Affiliations:** 1 Department of Climate & Environment, Norwegian Research Center (NORCE), Nygårdsporten 112, NO-5008 Bergen, Norway; 2 Vlaams Instituut voor de Zee, Flanders Marine Institute InnovOcean site, Wandelaarkaai 7, 8400 Oostende, Belgium; 3 School of Biological and Marine Sciences, University of Plymouth, Plymouth PL4 8AA, U.K.; 4 School of Health Professionals, University of Plymouth, Plymouth PL4 8AA, U.K.

**Keywords:** additives, bioaccessability, bioavailability, chemical pollutants, plastic pollution, vector effect

## Abstract

Microplastics (MPs, <5 mm in size) are a grave environmental concern. They are a ubiquitous persistent pollutant group that has reached into all parts of the environment — from the highest mountain tops to the depths of the ocean. During their production, plastics have added to them numerous chemicals in the form of plasticizers, colorants, fillers and stabilizers, some of which have known toxicity to biota. When released into the environments, MPs are also likely to encounter chemical contaminants, including hydrophobic organic contaminants, trace metals and pharmaceuticals, which can sorb to plastic surfaces. Additionally, MPs have been shown to be ingested by a wide range of organisms and it is this combination of ingestion and chemical association that gives weight to the notion that MPs may impact the bioavailability and toxicity of both endogenous and exogenous co-contaminants. In this mini-review, we set the recent literature within what has been previously published about MPs as chemical carriers to biota, with particular focus on aquatic invertebrates and fish. We then present a critical viewpoint on the validity of laboratory-to-field extrapolations in this area. Lastly, we highlight the expanding ‘microplastic universe’ with the addition of anthropogenic particles that have gained recent attention, namely, tire wear particles, nanoplastics and, bio-based or biodegradable MPs, and highlight the need for future research in their potential roles as vehicles of co-contaminant transfer.

## Introduction — the case for microplastics as a vehicle for chemicals

‘Microplastics’ (MPs) is used as a catch-all term to represent a complex variety of properties that arise during both the manufacturing process and following release into the environment [1]. Plastics are composed of different organic polymers to which an array of chemicals (termed here as ‘endogenous chemicals’, e.g. plasticizers, colorants, fillers and stabilizers) are added to enhance certain properties, such as rigidity, malleability, or thermal resistance and prolonging life [2]. Depending on use, plastics are produced within the MP size range of <5 mm (primary MPs such as microbeads) or into larger products that can subsequently breakdown releasing MPs (secondary MPs) [3,4]. MPs in the environment exist as a heterogeneous mixture of physical and chemical properties [1,4,5] and undergo several environmental transformations such as weathering, fragmentation, and biofilm and microbial colonization [6–8].

These transformations will affect how MPs interact within their environment — an environment that already contains a plethora of potential co-contaminants (termed here as ‘exogenous chemicals’). Tens of thousands of chemical entities are found on the global market with approximately 2000 new chemicals added each year [9]. The widespread ingestion of MPs, documented across aquatic taxa [10–12], provides a pathway for both endogenous and exogenous chemicals to enter the organism. Yet the role of MPs as chemical vectors not only relies upon the association with co-contaminants and the influence of the ambient environment, but considerations of the organism's biology and physiology are also important. For instance, feeding modes, gut retention times and digestive physiology will all play a role in whether the MP-associated chemical is bioaccessible and then bioavailable to the organism [5,13]. Toxicological consequences may result if the co-contaminants reach and become available at sites of biological activity.

**Figure 1.**
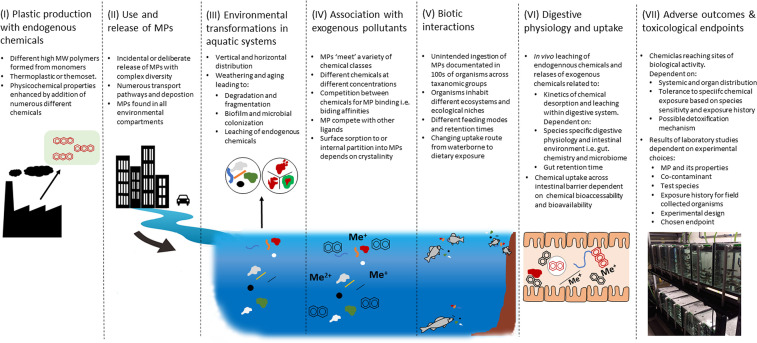
Schematic diagram of the complexity involved in the association of MPs with co-contaminants. Starting at the production stage where a range of endogenous chemical ‘additives’ (schematic aromatic rings in red) are incorporated into the polymeric resin (green) (I). MPs entering or produced through breakdown in the environment are a complex suite of physico-chemical properties (II) which are subject to the environmental transformations (III). MPs are known to sorb of exogenous pollutants, such as hydrophobic organic pollutants (schematic aromatic rings, black) and metals (Me^+^ and Me^2+^ black) (IV) and be ingested by biota (V). Within the digestive system endogenous additives (red) leach out of the MP and sorbed exogenous pollutants (black) desorb. The released chemicals may then be available for uptake (VI). The onset of toxicological outcomes will depend on the further transport of the chemicals to sites of biological activity and the ecotoxicological assessments of effects will depend on experimental choices made (VII). The complexity and array of variables presented here highlights why the role of MPs as chemical carriers is still debated. Schematic based on Khan et al. [5].

The role of MPs as chemical carriers has been the subject of much investigation, debate and speculation (see reviews [5,14–18]), but consensus remains elusive. The interactions of MPs, environment, chemicals and biota are summarized in Figure 1, and whilst laboratory studies can only investigate a portion of this complexity at one time, it is precisely this complexity that has made the role MPs in co-contaminant transfer one of the most studied and divergent topics in MP research.

## Biotic effects of microplastics and co-contaminant exposure

### Endogenous chemicals

During the manufacturing of plastics various substances often termed as ‘additives’ are combined with the polymeric resins to improve the properties of final applications and a few other reaction by-products will be further accidentally incorporated [2,19,20]. When plastic debris reach aquatic environments, these endogenous substances, some of which are known to be toxic to biota, can migrate from the resin to the external medium, as substances are often physically, rather than chemically, bonded to the main polymer matrix [20]. The leaching of substances from MPs may occur at higher rates compared with macroplastic litter due to their increased surface area to volume ratio. The majority of organic additives have a low hydrophobicity, or low octanol–water partition coefficient (*K*_ow_), and a low molecular mass [21]. Therefore, exposure of biota can be low due to the low diffusion of organic chemicals from the plastic to the water [21,22], as additives with a higher potential for toxicity and bioaccumulation have a higher *K*_ow_ [19].

Some endogenous chemicals found in aquatic environments are known to have toxicological properties, such as phthalates, bisphenol A (BPA), nonylphenol (NP) and brominated flame retardants (BFR) [19], as well as trace metals such as cadmium (Cd), lead (Pb), antimony (Sb) and tin (Sn) [2]. Of concern are the leaching rates of endogenous chemicals from weathered MPs [20], in particular when in gastrointestinal fluids during digestion where high levels of surfactants and lower pH may facilitate the migration process of compounds from the plastic resin [23]. Gut retention times of MPs vary amongst invertebrate species depending on physiology and relative MP size and shape and can last between a few hours and weeks. During this period, endogenous substances will increase in their bioaccessibility and bioavailability due to a low pH environment which enhances leaching from the polymeric matrix and further due to an affinity to fatty tissues of hydrophobic endogenous chemicals [22]. For example, Kühn et al. [24] demonstrated *in vitro* that environmental MPs will leach additives to stomach oil of northern fulmars. Tanaka et al. [25] demonstrated that feeding seabird chicks with MPs spiked in their resin with additives induced accumulation at 101–105 times above baseline in the tested individuals. However, a modeling exercise demonstrated that MPs may have a residual contribution to the accumulation and toxicity induced by leachates in the intestinal tracts of lugworms and in the North Sea cod when compared with sources of these contaminants such as water or sediments, but *in vivo* experimental validation is still required [22].

### Exogenous chemicals

MPs enter an environment that already contain a chemical cocktail, including hydrophobic organic contaminants (HOCs i.e. polycyclic aromatic hydrocarbons (PAHs) and polychlorinated biphenyls (PCBs)) [26,27], trace metals [28,29] and pharmaceuticals [30–32], all of which have been measured on MPs collected from the environment. These exogenous chemicals can ‘sorb’ (covering both surface adsorption and internal partition) to the MP based on the structure of the polymer (i.e. its ratio of crystalline and amorphous regions) [33]. The ingestion of the MPs can thus change the route of uptake compared with the dissolved form of the exogenous chemical from waterborne to dietary exposure [34] and following desorption within an organisms’ digestive system there is potentially a greater level of chemical exposure [35]. Accordingly, this so-called ‘vector-effect’ [3] has been the subject of much research and speculation, but even the earliest investigations demonstrated the varying nature of the vector phenomenon where MPs enhanced the bioavailability and toxicity of co-contaminants [36,37], where the addition of MPs to the exposure scenario resulted in negligible impacts [38], and cases where MPs reduced pollutant bioavailability [39].

The scientific literature in this area is too vast to comprehensively cover in this short review (see recent reviews [5,14–18]); however, recent descriptions of vector effects continue to vary. Two approaches are used to determine vector effects, (i) to directly measure chemicals in tissues to determine the influence of MPs to the exposure scenario and (ii) measure a toxicological marker of exposure as an indicator of chemical bioavailability and biological reactivity. Numerous laboratory studies have reported that exogenous contaminants sorbed to MPs are bioavailable and lead to a measurable transfer into the tissue [40–42] and toxicological impacts based upon the assessment of biomarkers [42–44], even if the tissue burdens did not correspondingly increase [45–47]. Conversely, several studies have demonstrated that at least for some measured endpoints, the role of MPs on chemical-induced negative impacts is not significant [48–52]. Using a novel ‘feeding tube’ method to directly introduce polyethylene and polystyrene MPs loaded with PCB-153 into the digestive tract of fish larvae showed no transfer of the PCB from MP into the tissue [53]. In the exposures of *Talitrus saltator* MPs were shown to carry HOCs into the tissue following ingestion, but when uncontaminated MPs were fed to sand hoppers, then the MP scavenged the chemicals and reduced the tissue burden [54]. Thus, under some circumstances, MP ingestion can potentially perform a ‘cleaning effect’ [54,55]. The transfer of PCBs from MPs under simulated gut fluid conditions was demonstrated to be biphasic and reversible [55].

The combination of MPs and co-contaminants may be viewed as similar to the interactions within a chemical mixture — independent or dependent action, or additivity, synergism or antagonism [56]. The joint exposure of MPs and the pharmaceutical triclosan to marine microalgae induced antagonistic effects with increasing MP concentrations reducing the triclosan toxicity based on the adsorption of the chemical to the plastic surface [57]. However, the co-exposure of the marine copepod *Acartia tonsa* to polyethylene microbeads and triclosan resulted in a relatively obscure mixture effect known as potentiation in which the MP, without itself being toxic, enhanced the toxicity of triclosan [56]. Thus, even when assessed through a recognized framework designed to disentangle the effects of single components within a mixture, the MP vector effect does not provide consistent outcomes. Though little used in MP research, the analysis of mixtures may provide a mechanistic insight into the individual roles of each competent within the MP-co-contaminant combination and further attention with this approach would be warranted.

### Focus on digestive physiology

Recognizing that there are important differences between all the studies described in the preceding sections that impact the outcome — choices relating to MP properties, co-contaminant, species, experimental design and biological endpoint (see Figure 1) — there remains disparity in descriptions of the MPs a chemical carrier which goes beyond the interactions and sorptive behavior in the test media and needs to consider physiology, particularly that of the intestinal environment. If MP ingestion is the assumed route of entry, then there are two possibilities for MPs and co-contaminants to enter the gastrointestinal tract (GIT) of aquatic animals — independently or with the co-contaminant associated (sorbed) to the MP [58]. In the GIT the fate of the MP and co-contaminant to remain independent, remain sorbed or desorb is largely driven by the gut lumen environment. Perhaps the earliest study to investigate HOC desorption in simulated gut conditions showed pH and temperature were important factors in determining desorption rates, suggesting that warm-blooded animals could be of the greater threat of MP-facilitated HOC transfer, but desorption also occurred in cold-blooded conditions representative of fish and invertebrates [59]. Recent follow-up studies have also demonstrated that both endogenous and exogenous chemicals separate from the MP within intestinal and biological fluids and conditions [13,60,61].

However, the lumen of the gastrointestinal tract (GIT) is a dynamic environment that varies between species and within species. For instance, the luminal pH of the polychaete worms *Lumbriculus variegatus* and *Arenicola marina* are 5.4–6.5 and 6.8–7.2, respectively, whereas carnivorous fish, such as rainbow trout (*Oncorhynchus mykiss*) exhibit a wider range pH 2.0–8.5 [62]. In the latter, the GIT is compartmentalized into different anatomical regions, with an acidic lumen in the stomach and alkali lumen in the intestinal regions. The pH is a main driver for determining the partitioning of chemicals onto the surface of MPs for ionizable organic chemicals [63] and dissolved metals [64,65], typically with lower pH values causing less chemical to bind to the surface of the MPs. For fish, at least, the variation in pH along the lumen of the GIT creates the potential for the cycling of chemicals on and off the MP [55]. Temperature, salinity and ionic strength have been shown to affect the sorption behavior of co-contaminants to MPs [15]. However, determining the relative contribution of each GIT parameter to the potential for vector effects and co-contaminant transfer is difficult *in vivo*, but a greater understanding of the role of species-specific digestive physiology is paramount to better understand the toxicological effects of MP and co-contaminant exposures.

## Laboratory-to-field extrapolation of MP co-contaminant studies

The ecotoxicological consequences of MPs and co-contaminants have largely been studied within laboratory settings. In extrapolating those findings to the natural world, two pertinent questions need to be addressed: (i) do laboratory studies realistically reflect the complexity of MPs in the environment and (ii) are MPs relevant chemical carriers compared with other potential sorbents? It is now established that in the environment, MPs are neither just pristine nor just contaminated, but rather exist in a continuum as a class of complex pollutants from different polymer types, shapes and sizes, at different levels of environmental transformations, and which can leach or sorb a multitude of chemicals (Figure 1) [1,5,18,66]. Despite this, most co-contaminant experimental studies employ aspects that lack environmental relevance; the use of pristine MPs, single polymers and MP types (e.g. the overuse of polystyrene spheres [67]) at levels above field concentrations coupled with single pollutants, short equilibrium times or methods to artificially hasten sorption kinetics [5,33]. Thus, studies with different MP morphologies are needed to reflect environmental prevalence [68] and as different types may exhibit different gut passage times which may affect chemical transfer from MP to tissue. Natural ageing (i.e. weathering) of MPs increases their adsorption affinity towards contaminants, but this parameter has seldom been considered in the effect assessments of MPs. The weathering of plastics in environmental settings is affected by exposure to UV radiation (sunlight), temperature shifts, humidity, and oxygen and ozone levels [69–71], and in turn the weathering of MPs can further play an important role in the leachates released and toxicity to organisms [20,72,73]. Furthermore, as climatic conditions shift due to global change (e.g. lower pH, increased temperature and fluctuating salinities) the impact of such parameters should be better linked to plastic pollution [66,74].

The aspect of relevance has been most comprehensively addressed by Koelmans et al. [22]. Briefly, the authors modeled analysis considered the whole mass of various compartments of the ocean including plastics, and then in which compartment exogenous HOCs may preferentially reside based upon partition coefficients. Ocean water would hold 98.3% of HOCs in the ocean and plastics just 0.0002% — in last place of the nine compartments included in the model [22]. Thus, when assessing the relative importance of MPs as chemical carriers, other compartments namely food and water, may be of greater importance as contaminant vectors; however, it is not possible to entirely disregard the link between MP ingestion and chemical availability [18]. Similarly, the transfer of endogenous chemicals is not accounted for.

## Expanding the microplastics universe

As the MP field progresses, new classes of anthropogenic particles are coming into focus and the same questions regarding chemical transfer are being asked. Nanoplastics (defined as <1 µm by ISO [75]) have been shown to be taken up by invertebrates [76,77] and translocated across the gastrointestinal membrane of fish in an *ex vivo* gut sac model [78]. Nano-sized particles have the potential to achieve cellular internalization via endocytotic mechanism and with this exists the possibility that endogenous and exogenous chemicals associated to nanoplastics may be carried into the cell. Coupled with the greater biological reactivity at the nano-size, the overall hazard of nanoplastics may be greater than MPs [79]. However, clear demonstrations of this potential are currently absent from the literature.

Concerns about plastic pollution and greater environmental sustainability have promoted ‘bioplastics’ as an alternative to conventional fossil-fuel-based polymers. The term ‘bioplastics’ may encompass both bio-based plastics made from renewable or natural sources (i.e. plant material) and biodegradable plastics that are made from materials which can be subject to enzymatic degradation of the polymeric matrix [20]. Thus, whilst conventional wisdom would say that bioplastics are designed to degrade faster than conventional plastics, there is specificity to the conditions of degradation, such as the right medium (water, soil, compost), and the absence of such conditions may result in a longer than expected residence time in the environment [80]. Though generally considered ‘green’ the bioplastic polyhydroxybutyrate (PHB) still contained a wide variety of exogenous chemicals and showed slight toxicity to sea urchin larvae [81]. Also using PHB as a test bioplastic, Magara et al. [82] compared the effects of polyethylene and PHB MPs as a vector of fluoranthene to *Mytilus edulis* with the two polymers exhibiting similar minimal differences to fluoranthene-only exposures. Thus, whether such materials constitute toxicologically safer alternatives is not yet verified as the literature is limited.

Tire wear particles (TWPs), tire and road wear particles (TRWPs), recycled crumb rubber (RTC) and tire-repair-polished debris (TRD) are rubber-related additions to the MP field [83–85]. Of these TWP is perhaps the most discussed with estimates of release suggesting that TWP is a significant component of MP pollution [86]. The chemicals added to tires during manufacturing have been shown to readily be released from the tire under laboratory conditions [87]. This complex ‘leachate’ has been shown to be toxic to a variety of aquatic organisms [87,88] with some specific chemicals now being pinpointed as known toxic agents. For instance, 6PPD-quinone was responsible for the acute toxicity of Pacific Northwest coho salmon observed in the field [89]. Recent studies with TWP have focussed on the particle and the leachate with several species ingesting TWP [84,90,91] and the two fractions showing distinct toxicities [91,92]. Thus, the role of the rubber particle delivering leachate *in vivo* requires greater attention.

## Conclusions

The role of MPs in effecting the bioavailability and toxicological consequences of endogenous and exogenous co-contaminants has been a much-debated aspect of plastic pollution. There is a wealth of in-depth literature on the subject (see reviews [2,5,15,16,18,19], but experimental studies often display inconsistencies. This is not surprising since the delivery of chemicals by MPs is dependent on multiple inter-connected factors (Figure 1) [5,47]). Thus, it remains difficult to judge whether MPs are realistic carriers of chemicals and furthermore, based on modeled analysis, whether MPs are relevant to study in this context given their relative contribution to oceanic mass compared with other sorbents [22]. The expansion of the field to include a greater range of particles, namely, nanoplastics, TWP and ‘bioplastics’, will increase the focus to cellular-level vector effects, leachate-related toxicity and ‘benign-by-design’, but future research should also consider the complex processes involved in MP-facilitated chemical transfer (Figure 1) with greater attention needed for biological parameters. Overall, greater environmental and physiological realism is needed to bridge the gap between the laboratory and the real world.

## Summary

- The transfer of endogenous or exogenous co-contaminants from MPs to biota is one of the most studied aspects of plastic pollution.
- Consensus as to the validity and relevance of MPs as chemical carriers is still debated.
- A multitude of inter-connected factors from production and release, environmental transformations to biological and physiological interactions need to be considered.
- Greater environmental realism is needed to bridge the gap between laboratory studies and the real world.
- New particles such as nanoplastics, TWPs and bioplastics expand the scope for chemical transfer.

## Abbreviations

GIT: gastrointestinal tract
HOCs: hydrophobic organic contaminants
MPs: microplastics
PCBs: polychlorinated biphenyls
PHB: polyhydroxybutyrate
TWPs: tire wear particles
